# *Candida glabrata* susceptibility to antifungals and phagocytosis is modulated by acetate

**DOI:** 10.3389/fmicb.2015.00919

**Published:** 2015-09-04

**Authors:** Sandra Mota, Rosana Alves, Catarina Carneiro, Sónia Silva, Alistair J. Brown, Fabian Istel, Karl Kuchler, Paula Sampaio, Margarida Casal, Mariana Henriques, Sandra Paiva

**Affiliations:** ^1^Centre of Molecular and Environmental Biology, Department of Biology, University of MinhoBraga, Portugal; ^2^Centre of Health and Environmental Research, School of Allied Health Sciences, Polytechnic Institute of PortoPorto, Portugal; ^3^Centre for Biological Engineering, University of MinhoBraga, Portugal; ^4^Institute of Medical Sciences – School of Medical Sciences, University of AberdeenAberdeen, UK; ^5^Department of Medical Biochemistry, Max F. Perutz Laboratories, Medical University of ViennaVienna, Austria

**Keywords:** *Candida glabrata*, acetate, transporters, phagocytosis, antifungal drug resistance, fluconazole, candidiasis

## Abstract

*Candida glabrata* is considered a major opportunistic fungal pathogen of humans. The capacity of this yeast species to cause infections is dependent on the ability to grow within the human host environment and to assimilate the carbon sources available. Previous studies have suggested that *C. albicans* can encounter glucose-poor microenvironments during infection and that the ability to use alternative non-fermentable carbon sources, such as carboxylic acids, contributes to the virulence of this fungus. Transcriptional studies on *C. glabrata* cells identified a similar response, upon nutrient deprivation. In this work, we aimed at analyzing biofilm formation, antifungal drug resistance, and phagocytosis of *C. glabrata* cells grown in the presence of acetic acid as an alternative carbon source. *C. glabrata* planktonic cells grown in media containing acetic acid were more susceptible to fluconazole and were better phagocytosed and killed by macrophages than when compared to media lacking acetic acid. Growth in acetic acid also affected the ability of *C. glabrata* to form biofilms. The genes *ADY2a, ADY2b, FPS1, FPS2*, and *ATO3*, encoding putative carboxylate transporters, were upregulated in *C. glabrata* planktonic and biofilm cells in the presence of acetic acid. Phagocytosis assays with *fps1* and *ady2a* mutant strains suggested a potential role of *FPS1* and *ADY2a* in the phagocytosis process. These results highlight how acidic pH niches, associated with the presence of acetic acid, can impact in the treatment of *C. glabrata* infections, in particular in vaginal candidiasis.

## Introduction

*Candida glabrata* is a prevalent human fungal pathogen that has become the second most frequent cause of candidiasis after *Candida albicans* in the United States, according to the SENTRY Program (Pfaller et al., 2001, 2011). Both species are commensal colonizers of mucosal surfaces. However, they are also successful opportunistic pathogens, causing either superficial or life-threatening disseminated infections reaching mortalities of around 40%. *C. glabrata* infections are especially difficult to treat due to a high inherent antifungal resistance, particularly against azoles (Pfaller, 2012). Moreover, *C. glabrata* colonizes host tissues as well as abiotic surfaces as biofilms (Silva et al., 2009). The formation of *Candida* biofilms raises important clinical issues because of a significant further increase in antifungal drug resistance as well as evasion of host immune defenses. Additionally, biofilm formation on medical devices can cause the failure of the device and can serve as a reservoir or source for future continuing infections (Douglas, 2003; Ramage et al., 2005; Calderone and Clancy, 2012).

Recently, it has been shown that *C. albicans* growth on an alternative carbon source, such as lactate, can affect stress adaptation, antifungal drug resistance, the cell wall architecture and proteome including immune detection (Ene et al., 2012a,b, 2013). Lactate-grown cells were taken up by macrophages less efficiently, but they were more efficient at killing and escaping phagocytosis (Ene et al., 2013). Of note, *C. glabrata* cells also resist killing by macrophages and have evolved distinct strategies for intracellular survival (Otto and Howard, 1976; Kaur et al., 2007; Seider et al., 2010). However, in these studies *C. glabrata* cells were grown in glucose. In the case of *C. albicans*, evidence exists that growth in the presence of an alternative carbon sources affects phagocytosis (Ene et al., 2013).

Adaptation to the host environment is essential for *Candida* spp. to infect and survive in various anatomical sites of the human host, some of which are clearly glucose-limited (Han et al., 2011). When *C. albicans* cells face a glucose-poor niche their metabolism changes. For instance, after phagocytosis of *C. albicans* by macrophages, repression of glycolysis occurs, the glyoxylate cycle, gluconeogenesis, and β-oxidation (Fradin et al., 2003, 2005; Prigneau et al., 2003; Lorenz et al., 2004; Barelle et al., 2006; Wilson et al., 2009) are induced. Additionally, data obtained with mutant strains in these pathways using mouse models of systemic candidiasis showed that the glyoxylate cycle is required for virulence (Lorenz and Fink, 2001, 2002; Barelle et al., 2006; Han et al., 2011), while β-oxidation is not (Piekarska et al., 2006). Thereby, it was postulated that acetyl-CoA feeding into the glyoxylate cycle might be derived from other non-fermentable carbon sources, such as lactate or acetate (Piekarska et al., 2006; Ramirez and Lorenz, 2007). This hypothesis was supported by the apparent expression regulation of two *C. albicans* carboxylic acids transporter genes, *JEN1* and *JEN2*, following macrophages internalization. *JEN1* encodes a monocarboxylic acid transporter (Soares-Silva et al., 2004) and *JEN2* a dicarboxylic acid permease (Vieira et al., 2010).

In *C. glabrata*, lactate assimilation is at least required for the survival in the intestine (Ueno et al., 2011). However, *JEN1* homologues are absent from the *C. glabrata* genome (Lodi et al., 2007). A transcription profile of *C. glabrata* following macrophage internalization revealed the up-regulation of genes involved in gluconeogenesis, β-oxidation and glyoxylate cycle, similar to what has been described for *C. albicans*. Moreover, a putative acetate transporter (*ADY2a*) appears to be induced (Kaur et al., 2007). The induction of *ADY2a* was also observed when *C. glabrata* is facing neutrophils (Fukuda et al., 2013), implying that in this environment, *C. glabrata* may assimilate acetate and other short-chain carboxylic acids for use as an alternative carbon source. Another observation supporting this hypothesis is that Fps1, a homologue of a *Saccharomyces cerevisiae* acetate channel, is also upregulated inside macrophages (Seider et al., 2010).

In *S. cerevisiae, ADY2* (also called *ATO1*) encodes a permease-mediating uptake of acetate–propionate–formate present in ethanol or acetate-grown cells (Casal et al., 1996; Paiva et al., 2004; Pacheco et al., 2012). In conjunction with its two additional homologues, *ATO2* and *ATO3*, it has also been implicated in ammonia export (Palkova et al., 2002; Vachova et al., 2009). Besides Ady2, Fps1 is a channel promoting the facilitated diffusion of undissociated acetic acid (CH_3_COOH) at low pH in *S. cerevisiae* (Mollapour and Piper, 2007). The *C. glabrata* genome encodes two putative homologues of *ScADY2* and one of *ScATO3*, all of which remain functionally uncharacterized. These *C. glabrata* homologues are CAGL0M03465g (designated *ADY2a*) and CAGL0L07766g (designated *ADY2b*), sharing 74 and 73% identity with *ScADY2*, respectively, and CAGL0A03212g (designated *ATO3*), sharing 62% identity with *ScATO3*. At least two *C. glabrata* homologues of *ScFps1* have been described: CAGL0C03267g (designated *FPS1*) and CAGL0E03894g (designated *FPS2*; Beese-Sims et al., 2012). These genes may act as glycerol channels, as the double mutant *fps1 fps2* accumulates glycerol, displays constitutive cell wall stress and hypersensitivity to treatment by caspofungin (Beese-Sims et al., 2012).

The aim of this study was to uncover how host microenvironments, and in particular, acidic niches that contain acetic acid, alter *C. glabrata* pathogenicity and susceptibility to antifungals. We have characterized acetic acid-grown planktonic and biofilm cells with respect to their antifungal drug resistance, as well as their interaction with macrophages. We examined growth at pH 5.0, a value above the pK*_a_* of the acid, where the charged anionic form (CH_3_COO^-^) acts as a nutrient rather than the undissociated acid (CH_3_COOH) conferring a weak acid stress (Casal et al., 2008; Stratford et al., 2013). Elucidating the effect of local nutrients and pH environment on drug resistance and phagocytosis can potentially provide new and effective treatment strategies for *C. glabrata* infections such as vaginal candidiasis.

## Materials and Methods

### Yeast Strains and Growth Conditions

*Candida glabrata* strains used in this work are listed in **Table 1**. Gene deletion was performed in the ATCC2001 background, 500 bp long homology flanking regions were amplified from genomic DNA, adding *Apa*I/*Xho*I RE sites, for the 5′ fragment, and *Sac*I/*Sac*II RE sites, for the 3′ fragment, and ligated into plasmid pSFS2 (Reuss et al., 2004). HTL strain was constructed from the ATCC2001 background, through the deletion of *HIS3, LEU2, TRP1*, using the SAT1 flipper method (Shen et al., 2005), and transformed by electroporation into *C. glabrata* ATCC2001 strain, as described elsewhere (Reuss et al., 2004). For construction of *CgFPS1* (CAGL0C03267g) and *CgADY2a* (CAGLOMO3465g) genomic deletion cassettes, the nourseothricin marker gene, *NAT1* was amplified from plasmid pJK863 (Shen et al., 2005), using primers fp_NAT1_U2 and rp_NAT1_D2. Barcodes and overlap sequences were added to the marker fragment and used to construct the disruption cassettes, by fusion PCR, as described previously (Noble and Johnson, 2005).

**Table 1:** *Candida glabrata* strains used in this study.

*C. glabrata* strains	Genotype	Reference
ATCC2001 (CBS138)	Wild type	ATCC collection (available at www.atcc.org)
HTL	Derived from ATCC2001, *his3*::FRT, *leu2*::FRT and *trp1*::FRT	Jacobsen et al. (2010)
*ady2a* (CAGL0M03465g)	Derived from HTL, *ady2a*::NAT1	Schwarzmuller et al. (2014)
*fps1* (CAGL0C03267g)	Derived from HTL, *fps1*::NAT1	Schwarzmuller et al. (2014)

The cultures were maintained on plates of YPD: yeast extract (1%, w/v), peptone (1%, w/v), glucose (2%, w/v) and agar (2%, w/v). For growth phenotypes yeast cells were grown at 30, 37, and 42°C during 96 h in synthetic complete (SC) media (prepared with 0.67% (w/v) Difco yeast nitrogen base mineral medium without amino acids plus 2 g/l complete amino acid mixture) with: glucose (2% w/v), lactic acid (0.5% v/v; pH 5.0), acetic acid (0.5% v/v; pH 5.0 or pH 6.0), citric acid (1%, w/v; pH 5.0) malic acid (1% w/v; pH 5.0), pyruvic acid (1% w/v), and succinic acid (1% w/v; pH 5.0), as sole carbon and energy sources and 2% (w/v) agar.

### Minimal Inhibitory Concentration (MIC) Determination

The MIC assays were performed by the microdilution method according to the Clinical and Laboratory Standards Institute (CLSI, 2008) document M27-A3, with some modifications, using RPMI 1640 broth, supplemented with 0.165 M of MOPS, with or without adding 0.5% acetic acid at pH 5.0 (Pfaller et al., 2008). Different concentrations of fluconazole ranging from 0 to 1250 μg/ml were used. The MIC of the antifungal agents against each *C. glabrata* strain was determined visually and by total number of colony forming units (CFUs). For this purpose each condition was serially diluted in 1× phosphate buffered saline (PBS) and 10 μl of cell suspensions of each dilution were plated in YPD medium. All the experiments were performed in triplicate, at least in three independent assays.

### Biofilm Formation and Antifungal Susceptibility

Biofilm formation was performed as described previously by Silva et al. (2009). Briefly, 200 μl of 1 × 10^5^ cells/ml suspensions in RPMI 1640 with or without 0.5% (v/v) acetic acid at pH 5.0 were placed into 96-wells polystyrene microtiter plates (Orange Scientific, Braine-l′Alleud, Belgium) and incubated at 37°C with gentle agitation. After 24 h, the entire medium was replaced by 200 μl of fresh medium. To study the effect of fluconazole on biofilm formation, different concentrations of fluconazole (312.5 and 1250 μg/ml) were prepared in RPMI 1640 medium (Sigma, St. Louis, MO, USA) and added to the 24-h old pre-formed biofilms. The microtiter plates were incubated for an additional 24 h.

### Biofilm Characterization

#### Biofilm Biomass Quantification

Total biofilm biomass was quantified by crystal violet (CV) staining (Silva et al., 2009). The culture medium was removed by aspiration and the biofilm washed once with 200 μl of PBS to remove non-adherent cells. The biofilms were fixed with 200 μl of methanol for 15 min. The microtiter plates were allowed to dry at room temperature, and 200 μl of CV (1% v/v) were added to each well and incubated for 5 min. The wells were then gently washed twice with water, followed by addition of 200 μl acetic acid (33%, v/v) to release and dissolve the stain. The absorbance was determined in a microtiter plate reader (Bio-Tek Synergy HT, Izasa) at 570 nm. The results are presented as percentage of biomass reduction. Experiments were performed at least three times using independent biological samples.

#### Biofilm Viability Quantification

The number of cultivable cells in biofilms was determined by the enumeration of CFUs counts. For that, the medium was aspirated and the biofilms washed once with PBS to remove non-adherent cells. Next, biofilms were scraped from wells and the suspensions were vigorously vortex-mixing for 2 min to disaggregate cells from matrix. Serial 10-fold dilutions in PBS were plated onto YPD plates and incubated for 24 h at 37°C. The results were presented as percentage of CFU reduction (log scale). The complete biofilm dispersal was confirmed by CV staining. Experiments were performed in duplicate and at least three times using independent biological samples.

#### Biofilm Structure

Biofilm structure was assessed by scanning electron microscopy (SEM). Biofilms were allowed to form in 24-wells polystyrene microtiter plates (Orange Scientific, Braine-l′Alleud, Belgium), each well containing 1 ml of 1 × 10^5^ cells/ml suspensions, as described previously. After 48 h of formation, biofilms were washed with PBS and dehydrated with alcohol (using 70%, v/v ethanol for 10 min, 90%, v/v ethanol for 10 min, and 100%, v/v ethanol for 20 min) and then air-dried. Prior to inspection, the base of the wells were mounted onto aluminium stubs, sputter coated with gold and observed with an S-360 scanning electron microscope (Leo, Cambridge, MA, USA).

### Gene Expression Analysis

Biofilms were grown as described above. After their formation, the medium was aspirated and the wells washed with PBS to remove the non-adherent cells. Biofilms were then scraped from wells and sonicated (Ultrasonic Processor, Cole-Parmer) for 30 s at 30 W. Cells were harvested by centrifugation at 8000 *g* for 5 min at 4°C. Additionally, planktonic cells, cells were grown in 25 ml of RPMI 1640 with or without 0.5% v/v acetic acid at pH 5.0 and in the absence or in the presence of 50 μg/ml fluconazole for 48 h at 37°C.

For total RNA isolation and purification, we used the E.Z.N.A.^®^ Total RNA Kit, Omega Bio-tek^®^ according to the manufacturer’s instructions. To remove residual DNA contaminations, samples were treated with DNase I (Invitrogen^TM^), according to the manufacturer’s instructions. Purity and concentration of total RNA was evaluated using a NanoDrop spectrophotometer. To synthesize the cDNA the High-Capacity cDNA Reverse Transcription Kit (Invitrogen^TM^) was used according to the manufacturer’s instructions.

#### Quantitative Real-time PCR

Relative quantitative RT-PCR of the cDNA samples was carried out in a CF X96 Real-Time PCR System from Bio-Rad Laboratories using Power SYBR^®^ Green PCR Master Mix (Applied Biosystems^®^). The primers used to amplify the selected genes using qPCR were designed using Primer Blast (Rozen and Skaletsky, 2000; Ye et al., 2012) and are listed in **Table 2**. The reaction mixture was set up in a total volume of 20 μl using 10 μl of SYBR^®^ Green PCR Master Mix, 0.3 μM of each primer and 4 μl of cDNA (diluted 1:20) and nuclease-free water. Thermo cycling conditions for qPCR were 10 min at 95°C, followed by 40 cycles of 95°C for 15 s, the correspondent annealing temperature for each primer for 1 min (Supplementary Table S1), and 65°C for 5 s. A negative control without template was conducted for each gene in each PCR run, and a control for DNA contamination was implemented by using the purified RNA samples as template. The housekeeping gene, PGK1 (Li et al., 2012) was used to normalize the gene expression. The relative quantification of gene expression was performed by the ΔCT method (Livak and Schmittgen, 2001). Experiments were performed in two independent biological samples.

**Table 2:** List of primers used in this study.

Target gene	Primer	Sequence (5′→3′)	PCR product size (bp)
CAGL0C03267g	FPS1 fwd FPS1 rev	CATGTCTACCGCTGCTGCTA AGTGGCCTAGGTTCAACACG	113
CAGL0E03894g	FPS2 fwd FPS2 rev	TGCTAGAAGCCGCAGACAAA GCCTCCAAGACCGTCGTTAT	88
CAGL0M03465g	ADY2a fwd ADY2a rev	TGTGCTCCTACGGTGGTTTC GGTCCAGCCCAGTAGGTAGA	131
CAGL0L07766g	ADY2b fwd ADY2b rev	CCCCACCATCGTCTCACAAA AGCAGCAGGACCGACTACTA	140
CAGL0A03212g	ATO3 fwd ATO3 rev	CGTGAGCAACATACCCCAGT CAAGGACAGCACAAGGCAAC	93
Housekeeping gene CAGL0L07722g	PGK1 fwd PGK1 rev	CAAACGGTGAAAGAAACGAGAA CCGACACAGTCGTTCAAGAAAG	100

### Phagocytosis Assays

The murine macrophage-like cell line J774A.1 was cultured in complete Dulbecco’s modified eagle’s medium (DMEM) at 37°C in a 5% CO_2_ atmosphere. DMEM was supplemented with 10% heat-inactivated fetal bovine serum (FBS) (Valbiotech), 2 mM L-glutamine, 1 mM sodium pyruvate, and 10 mM HEPES. After reaching confluent growth, macrophages were recovered, washed, resuspended in complete DMEM, and seeded in a 96-wells plate at 1 × 10^5^ macrophages/well (final volume 250 μl) in triplicates. Cells were then incubated overnight at 37°C in a 5% CO_2_ atmosphere, to allow for macrophage adherence. In the following day, phagocytic cells were washed two times with PBS buffer to remove non-adherent cells. Yeast cells grown for 48 h at 37°C in RPMI 1640 medium, with or without 0.5% acetic acid, were recovered by a brief centrifugation step, and washed twice in sterile 1× PBS. Yeast suspensions were added to each well at MOI of 1M:5Y (Macrophage:Yeast cells) at 37 °C and 5% CO_2_. After co-incubation with macrophages (3 and 18 h), plates were centrifuged at 1000 *g* for 2 min and 150 μl of supernatant was stored at -80 °C for subsequent Tumor Necrosis Factor Alfa (TNF-α) quantification. To induce macrophages lysis, 150 μl of cold H_2_O with 10% saponin were added to macrophage cultures. After carefully mixing, serial dilutions were prepared and 10 μl of the two higher dilutions were plated in quadruplicated in YPD agar. After 24 h of incubation at 37°C, the number of viable yeast cells was determined by CFU-counting. Controls consisting of yeast cells grown in the same conditions without macrophages were added. The percentage of yeast cells killing by macrophages were calculated according to the following equation:

$$\%\;\mathrm{yeast}\;\mathrm{killing}=\frac{\mathrm{CFU}\;\mathrm{of}\;\mathrm{control}\;\mathrm{well}-\mathrm{CFU}\;\mathrm{of}\;\mathrm{test}\;\mathrm{well}}{\;\mathrm{CFU}\;\mathrm{of}\;\mathrm{control}\;\mathrm{well}}\times 100$$

Two independent experiments of this assay were performed.

Phagocytosis was also examined by microscopy and cytometry according to a recently described protocol (Carneiro et al., 2014). Briefly, yeast cells grown for 48 h at 37°C in RPMI 1640 medium, with or without 0.5% acetic acid, were recovered by centrifugation and washed twice in sterile PBS. Yeast cells were incubated for 10 min in formol/ethanol (1:9), to fix cell wall pathogen-associated molecular patterns (PAMPs); cells were then washed five times with PBS for a complete removal of formol/ethanol. Yeast cells were incubated with Sytox-Green (Invitrogen) at 1 μM during 10 min, at room temperature in the dark, washed with PBS, to remove unbound dye, and brought to the desired cell density in complete DMEM.

Culture of murine macrophage-like cell line J774A.1 was performed as described above. After reaching confluency, macrophage cells were recovered, washed, and resuspended in complete DMEM at a final density of 5 × 10^5^ cells/ml. For flow cytometry, 3 ml of the cell suspension were transferred to a 6-wells tissue culture plates. For fluorescence microscopy assays, 1 ml cell suspension aliquots were transferred to 24-well culture plates containing clean sterile glass coverslips (diameter 13 mm). Macrophage cells were incubated overnight, at 37°C in a 5% CO_2_ atmosphere, to allow for macrophage adherence. In the next day, macrophages cells were washed two times with PBS to remove non-adherent cells and the adhered cells were used for phagocytic assays.

Macrophages were incubated with yeast cells suspensions previously labeled with Sytox-Green at MOI of 1M:5Y for 30 min at 37°C and 5% CO_2_. After incubation, plates were kept on ice to stop phagocytosis, then washed twice with complete DMEM to remove unbound yeasts, and finally resuspended in 1 ml of the same medium. Macrophages and adhered yeasts were then incubated with propidium iodide (Sigma–Aldrich) at a final concentration of 120 μg/ml for 5 min at room temperature (Carneiro et al., 2014). Cells were analyzed by flow cytometry (EPICS XL-MCL, Beckman-Coulter Corporation) and by fluorescence microscopy (Leica DM5000B). Results were obtained from two replicates of each strain/condition. Cytometry results were analyzed using the Flowing Software Version 2.5.1. These experiments were performed in duplicates.

### Cytokine Quantification

TNF-α production by macrophages infected with *C. glabrata* strains was measured using the mouse TNF-α ELISA ReadySETGo Kit (eBioscience, San Diego, CA, USA), according to the manufacturer’s instructions. Supernatants recovered from the CFU assay stored at –80°C were used as samples. Macrophages without yeast cells supernatants were used as a negative control, and macrophages infected with *Escherichia coli* cells were used as a positive control (LPS control). These experiments were performed in triplicate.

### Statistical Analysis

Results were analyzed using GraphPad Prism 5. The statistic tests used were one-way ANOVA with Turkey multiple comparison test. All tests were performed with a confidence level of 95%.

## Results

### Acetic Acid Increases the Susceptibility of *C. glabrata* Planktonic Cells to Fluconazole

*Candida glabrata* ATCC 2001 planktonic cells’ susceptibility to antifungal drugs was determined by the microdilution method and confirmed by CFU-counting for two different growth conditions: RPMI and RPMI-containing 0.5% acetic acid, both at pH 5.0.

In RPMI medium, we obtained a MIC value of 150 μg/mL for *C. glabrata* cells and a minimal fungicidal concentration (MFC) value of 312.5 μg/ml (**Table 3**). In acetic acid medium, we obtained lower fluconazole MIC and MFC values (50 and 150 μg/ml, respectively; **Table 3**). These results show that acetic acid enhances the susceptibility of *C. glabrata* cells to fluconazole.

**Table 3:** Effect of fluconazole in wild type (WT) *C. glabrata* ATCC 2001 planktonic cells using two different growth conditions: cells grown in RPMI pH 5.0 and cells grown in RPMI 0.5% acetic acid pH 5.0.

Condition	MIC (μg/ml)	MFC (μg/ml)
RPMI pH 5.0	150	312.5
RPMI 0.5% acetic acid pH 5.0	50	150

*The minimal inhibitory concentrations (MICs) were determined visually and the minimal fungicidal concentrations (MFCs) by total number of CFUs.*

### *Candida glabrata* Susceptibility to Fluconazole is Higher in Biofilms than in Planktonic Cells

**FIGURE 1:**
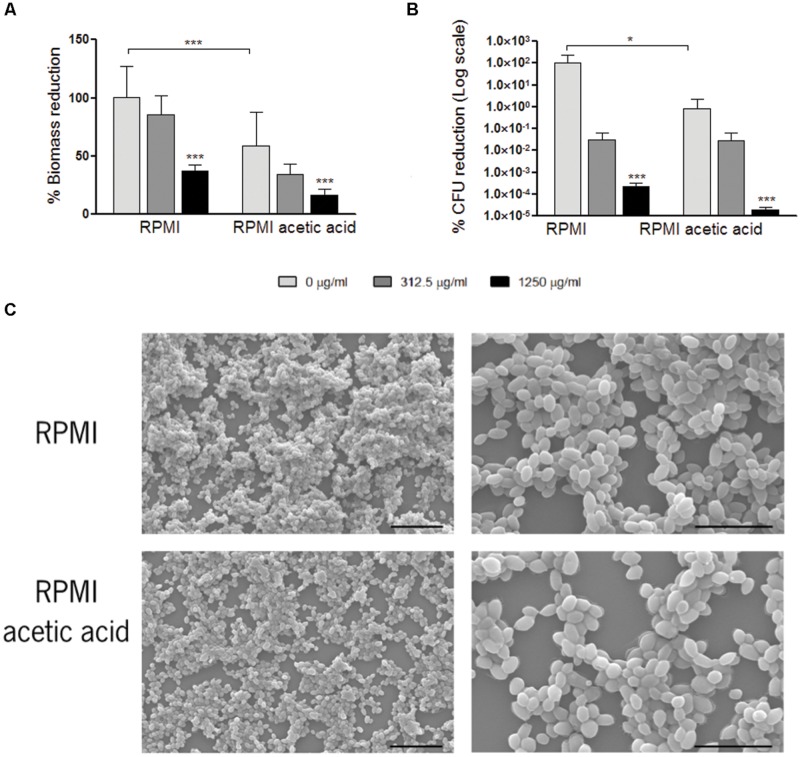
**Effect of fluconazole in *Candida glabrata* ATCC2001 pre-formed biofilms.** Graph **(A)** represents the percentage of biomass reduction that was quantified by crystal violet staining and **(B)** the percentage of colony-forming units (CFUs) reduction. Two conditions were tested: RPMI pH 5.0 and RPMI 0.5% acetic acid at pH 5.0. Error bars represent SD. ∗ and ∗∗∗ means that results are statistically significant (*p* < 0.05) and (*p* < 0.001), respectively. **(C)** Scanning electron microscopy of *C. glabrata* 48 h formed biofilms grown in RPMI pH 5.0 and RPMI 0.5% acetic acid pH 5.0. The left and right images in each media represent a magnification of 1000× and 3000×, respectively. Bars in the images correspond to 20 μm in 1000× magnification and 10 μm in 3000× magnification.

The experiments described above for *C. glabrata* planktonic cells were reproduced for *C. glabrata* biofilm cells. We compared *C. glabrata* biofilms obtained in a media with or without acetic acid (RPMI vs. RPMI with 0.5% acetic acid, pH 5.0; **Figure 1**). In the presence of acetic acid, biofilm biomass as well as viable cell numbers were reduced, when compared to RPMI medium only (**Figure 1**). In both conditions, aggregates of yeast cells were observed in the biofilms. However, in the presence of acetic acid these aggregates were smaller (**Figure 1C**).

To test the effect of fluconazole, RPMI media with or without addition of acetic acid at pH 5.0 was used for the growth of *C. glabrata* biofilms. After 24 h, fluconazole was added to the pre-formed biofilms and the total biofilm biomass and CFUs were evaluated. The presence of fluconazole did not result in the eradication of the biofilms, in any of the growth conditions tested. For both conditions, a statistically significant reduction in biofilm biomass and in CFU count was only obtained with the highest fluconazole concentration of 1250 μg/ml (**Figures 1A,B**).

### Gene Expression Profile in Planktonic and in Biofilm Cells

The use of acetic acid as a sole carbon and energy source implies that *C. glabrata* cells have specific transporters mediating acetate uptake. Hence, we assessed by qRT-PCR the expression profile of *C. glabrata* putative acetate transporters encoding genes *ADY2a, ADY2b, ATO3, FPS1*, and *FPS2*, both in planktonic and biofilm cells grown in RPMI with and without acetic acid.

**FIGURE 2:**
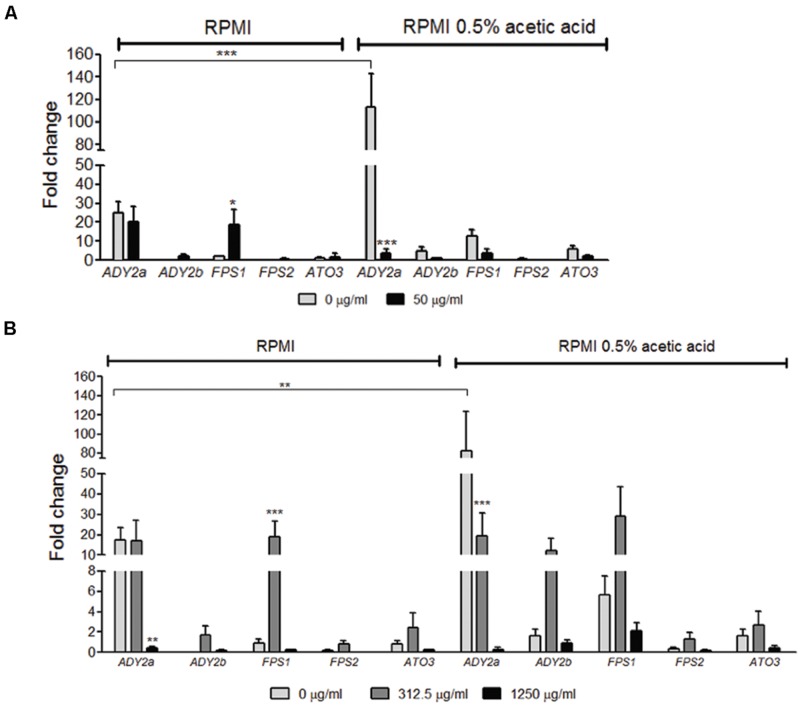
**Effect of acetic acid and fluconazole in the expression of *ADY2a, ADY2b, FPS1, FPS2*, and *ATO3* in *C. glabrata* ATCC 2001 planktonic **(A)** and biofilm cells **(B)**.** Cells and biofilms were grown in two different conditions: RPMI pH 5.0 and RPMI 0.5% acetic acid at pH 5.0. Legend displays fluconazole concentrations used. Gene expression was calculated using ΔCT method and normalized to PGK1 gene. Error bars represent SD. ^∗^, ^∗∗^, and ^∗∗∗^ means that results are statistically significant (*p* < 0.05), (*p* < 0.01), and (*p* < 0.001), respectively.

In the absence of fluconazole, *ADY2b, FPS1, FPS2*, and *ATO3* genes displayed a very weak level of expression in RPMI medium, both in planktonic and biofilm cells. However, the gene *ADY2a* was strongly induced in these conditions (**Figures 2A,B**). When cells were grown in the presence of acetic acid, all genes displayed a higher level of expression, except for *FPS2*, which was expressed at low levels. This increase was observed in both planktonic and biofilm cells (**Figures 2A,B**) and it was very striking for the *ADY2a* gene (*p* < 0.001 and *p* < 0.01, respectively).

The effect of the addition of fluconazole in the expression of the selected genes was also determined. In planktonic cells grown in RPMI with 50 μg/ml fluconazole, *FPS1* expression increased (*p* < 0.05) and *ADY2a* expression was maintained, in comparison with media without the drug. However, when planktonic cells were grown in the presence of acetic acid with 50 μg/ml fluconazole, a decrease in *ADY2a* (*p* < 0.001) expression was observed (**Figure 2A**).

The drug effect was also studied for biofilm cells in the presence of either 312.5 or 1250 μg/ml fluconazole. The response of these genes was dependent on the concentration of the drug and a clear pattern of expression was found: an increase with 312.5 μg/ml of fluconazole and a decrease with the highest drug concentration (1250 μg/ml). This behavior was identical for cells grown either in the presence or absence of acetic acid. The only exception was found for *ADY2a*, which displayed either a maintenance of expression or a reduction (*p* < 0.001) in the presence of 312.5 μg/ml fluconazole, both in RPMI and RPMI acetic acid, respectively.

### Biofilm Formation of *C. glabrata* in the Presence of Fluconazole

Considering that *ADY2a* and *FPS1* were the most strongly upregulated genes in acetate and given that *FPS1* expression was significantly affected by the presence of fluconazole, we selected these genes for further studies. The phenotype of *ady2a* and *fps1 C. glabrata* mutant strains (Schwarzmuller et al., 2014) was evaluated regarding their ability to grow in different carbon sources, at 30, 37, and 42°C. The carbon sources tested included glucose, glycerol, lactic, acetic, citric, malic, pyruvic, and succinic acids. All mutant strains had a growth profile identical to the wild type (WT) strain in all carbon sources tested (data not shown). Since acetate was used by the mutants as the only carbon and energy source, we accessed biofilm formation in the presence and absence of acetate (Supplementary Figure S1). Notably, biofilms obtained for the mutants and for the WT strains were similar, and all strains displayed increased biofilm biomass in media without acetic acid.

The effect of fluconazole was then tested in mutant biofilms in the presence and in the absence of acetic acid. For both conditions, a statistically significant reduction in biofilm biomass and in CFU count was observed with the highest concentration of fluconazole. This was a similar result to the one found for the WT strain. Moreover, no statistically significant differences were observed between mutant and WT strains.

### The Carbon Source Affects *C. glabrata* Interaction with Phagocytic Cells

Since our data suggested that cells grown in media containing acetic acid are more susceptible to fluconazole, we evaluated how macrophages interacted with *C. glabrata* cells previously grown either in RPMI or RPMI with acetic acid.

To evaluate macrophage phagocytosis of *C. glabrata* cells grown in the presence or absence of acetic acid, we used a previously described assay (Carneiro et al., 2014). This method allows the identification of four different macrophage populations by differential staining, based on the distinct interaction between macrophages and *C. glabrata* cells. Therefore, macrophages with only internalized *C. glabrata* cells (Sytox green-stained), with only surface adhered cells (PI stained), with both internalized and surface adhered cells (PI and Sytox green double stained) or with no contact with *C. glabrata* cells (Sytox green and PI negative staining) were clearly distinguished.

**FIGURE 3:**
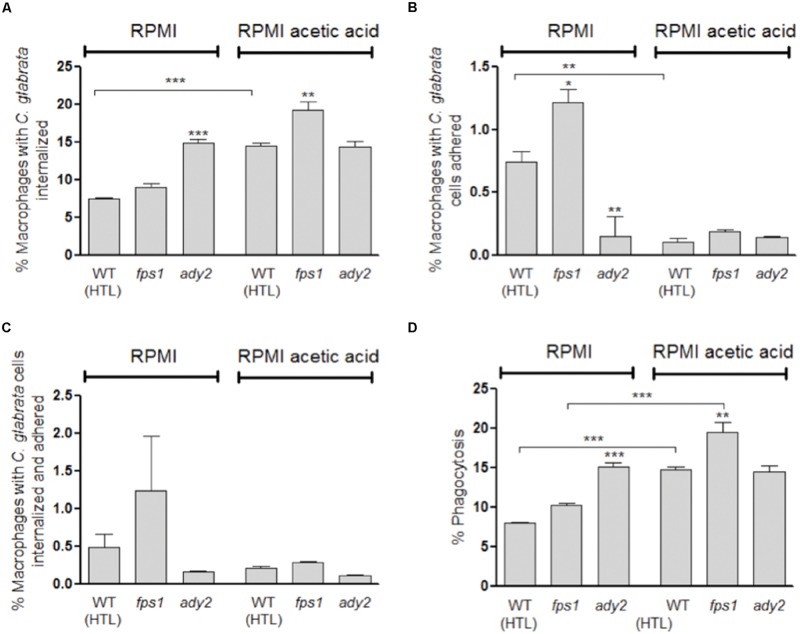
**Evaluation of phagocytosis of *C. glabrata* HTL, fps1, and ady2a cells by flow cytometry after 30 min of incubation at 37 °C with macrophages.** Before the phagocytosis assay yeast cells were grown in two different conditions: RPMI pH 5.0 and RPMI 0.5% acetic acid at pH 5.0. Graph **(A)** represents the percentage of macrophages with yeast cells internalized, **(B)** shows the percentage of macrophages with yeast cells adhered to their surface, **(C)** corresponds to the percentage of macrophages with yeast cells internalized and adhered, and **(D)** displays the percentage of phagocytosis, resulting from the sum of **(A)** and **(C)**. Error bars represent SD. ^∗^, ^∗∗^, and ^∗∗∗^ means that results are statistically significant (*p* < 0.05), (*p* < 0.01), and (*p* < 0.001), respectively.

Representative fluorescence microscopy and flow cytometry analyses are shown in Supplementary Figures S2 and S3. In general, upon 30 min of co-incubation, flow cytometry analysis showed that a higher percentage of macrophages with internalized yeast cells are present in comparison with the percentage of macrophages with adherent or, adherent and internalized yeast cells (**Figure 3**). Furthermore, *C. glabrata* WT cells, previously adapted to acetic acid, resulted in 14.5% of macrophages with internalized yeast cells which represents twice of what was found when WT cells were grown only in RPMI (7.6%, *p* < 0.001; **Figure 3A**). In addition, the percentage of phagocytosis index was also significantly higher (14.7%, *p* < 0.001) in WT acetic acid-adapted cells in comparison with *C. glabrata* cells grown without the acid (8.1%; **Figure 3D**).

We also assessed the capacity of macrophages to phagocytose *C. glabrata ady2a* and *fps1* mutant cells pre-grown in RPMI with or without acetic acid. A clear difference between the behavior of the different mutants and the WT cells was found (**Figure 3**).

Regarding *fps1* mutant cells, no significant difference in the phagocytosis index was observed in cells grown in RPMI in comparison with WT cells. Conversely, when adapted to acetic acid a higher phagocytosis index for this mutant was observed in comparison with the WT (*p* < 0.01; **Figure 3D**). These results suggest that adaptation to acetic acid renders *fps1* mutant cells more susceptible to macrophage phagocytosis.

This tendency was, however, not observed for *ady2a* mutant yeast cells, as in RPMI medium they were more susceptible to macrophage phagocytosis (*p* < 0.001), in comparison with the WT. In addition, in this medium, *ady2a* mutation induced a significant lower percentage of macrophage adherent yeast cells (*p* < 0.01, **Figure 3B**) and higher percentage of internalized yeast cells (*p* < 0.001, **Figure 3A**). These results suggest that *ady2a* mutation somehow enhances yeast cells recognition and engulfment by macrophages.

The phagocytosis index of acetic acid adapted *ady2a* cells was identical to the value found when cells were grown in sole RPMI medium. This was in contrast to what was observed for WT and *fps1* mutant cells, which exhibit higher susceptibility to macrophage recognition and engulfment, in RPMI acetic acid medium (**Figure 3**).

**FIGURE 4:**
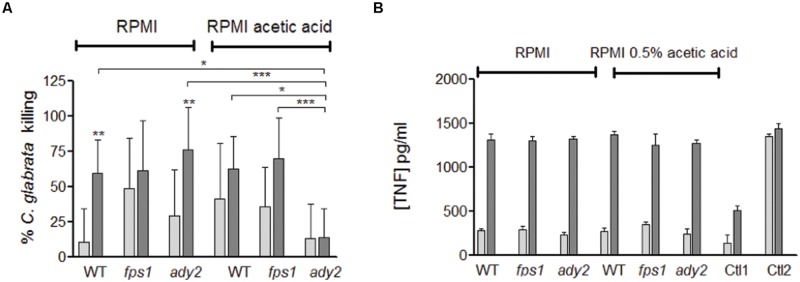
**Evaluation of the interaction of *C. glabrata* HTL, fps1, and ady2a cells with macrophages. (A)** Percentage of *C. glabrata* killing by macrophages evaluated by CFU and **(B)** the TNF-α concentration released by macrophages infected with *C. glabrata* strains in pg/ml, Ctl1 and Ctl2 corresponds to the macrophages control and incubated with LPS, respectively. The results were assessed at 3 and 18 h post-infection (white and grey bars, respectively). Before the phagocytosis assay yeast cells were grown in two different conditions: RPMI pH 5.0 and RPMI 0.5% acetic acid at pH 5.0. Error bars represent SD. ^∗^, ^∗∗^, and ^∗∗∗^ means that results are statistically significant (*p* < 0.05), (*p* < 0.01), and (*p* < 0.001), respectively, when comparing 3 with 18 h of incubation. Other comparisons are indicated by a bar.

To evaluate the effect of acetic acid adaptation on phagocyte interaction, macrophages were infected with *C. glabrata*, previously grown in the presence or absence of acetic acid, and yeast killing was assessed by CFU after 3 and 18 h of co-incubation. Additionally, TNF-α was quantified to evaluate the ability to induce a pro-inflammatory response. Results showed that the percentage of *C. glabrata* WT killed by macrophages was higher when cells were previously grown in RPMI with acetic acid in comparison with RMPI alone, in particularly after 3 h of incubation (**Figure 4A**).

Regarding *C. glabrata* mutant strains, although no statistical significance was reached, both *fps1* and *ady2a* cells appear to be more susceptible to macrophage killing than WT cells, when grown in RPMI media, in particularly at the early time point (**Figure 4A**). However, for yeast cells grown in the presence of acetic acid, *fps1* mutant behaved similarly to WT cells, while *ady2a* cells were more resistant to killing. The percentage of killing did not change with longer incubation periods but was significantly lower than WT or *fps1* strains, in the presence of acetic acid, for *ady2a* (WT vs. *ady2a, p* < 0.05 and *fps1* vs. *ady2a, p* < 0.001). All cells and conditions were able to trigger pro-inflammatory responses, with significant differences in the amount of TNF-α released from 3 to 18 h of incubation. The amount of this cytokine was similar in all conditions.

Taken together, these results show that WT *C. glabrata* cells, grown in the presence of acetic acid, are better internalized and killed by macrophages and suggest that acetic acid renders *ady2a* mutant more resistance to macrophage killing.

## Discussion

*Candida glabrata* has the ability to survive within different host niches, with diverse nutrient availability. In such niches, for example inside phagosomes, it faces glucose-limited environments. To survive, it has to change its metabolism and utilize non-fermentable carbon sources (Kaur et al., 2007; Roetzer et al., 2010; Fukuda et al., 2013). In the intestine, where glucose is scarce, *C. glabrata* requires Cyb2 for adaptation and survival (Ueno et al., 2011). This protein is required for the assimilation of lactate, a non-fermentable carbon source present in the intestine (Ueno et al., 2011). Other weak organic acids besides lactate are present in the intestine, namely acetate, which can be produced by intestinal microbiota (Yamaguchi et al., 2005). Moreover, this carboxylic acid is also present in other human niches, including the vaginal mucosa (Preti et al., 1979) and vaginal secretions (Huggins and Preti, 1976).

Ene et al. (2012a) showed that *C. albicans* cells grown in an alternative carbon source, lactic acid, were more resistant to amphotericin B but more susceptible to miconazole. Another study reported that acetic acid, other alternative carbon source, acted synergistically with clotrimazole and flucytosine in *C. glabrata* (Costa et al., 2013). In the present work, we show that acetic acid increases susceptibility to fluconazole in planktonic cells (**Table 3**). This finding has an important clinical impact, namely in the treatment of vaginal candidiasis, since this niche has an acidic pH and is rich in alternative carbon sources, such as lactic and acetic acid. In fact, Moosa et al. (2004) showed that fluconazole was fungicidal for *C. albicans* in a synthetic vagina-simulative medium with an acidic pH. The analysis of the components of the medium indicated that acetic acid was responsible for the synergistic, fungicidal effect (Moosa et al., 2004). This study also reported that two independent clinical isolates of *C. glabrata* were not killed when grown in YNB medium supplemented with acetic acid and fluconazole, contrary to what was observed for *C. albicans* strains (Moosa et al., 2004). By contrast, our work shows that the synergistic effect observed for *C. albicans* is also seen in *C. glabrata*.

The present work demonstrates that *C. glabrata* forms biofilms in media containing acetic acid (RPMI 0.5% acetic acid pH 5.0). However, in this media the biofilm is weaker in terms of total biomass and CFU when comparing to biofilms formed in the absence of acetate (**Figure 1**). In addition, we studied the effect of fluconazole in *C. glabrata* pre-grown biofilms in the presence and in the absence of acetic acid. Our results show that a statistically significant reduction in biofilm biomass and in CFU count is obtained only to the highest concentration of fluconazole, for both tested conditions (Supplementary Figure S1). These results are consistent with reports for *C. albicans* biofilms (Baillie and Douglas, 1998; Chandra et al., 2001a,b; Ramage et al., 2001), showing that biofilms needed about 10 to 100 times more quantity of antifungal drug to be eradicated when compared to planktonic cells.

Host defenses against *C. albicans* infection are characterized by a dynamic relationship between the activation of immune responses and the capacity of the pathogen to modulate these responses. The first step in the activation of the host immunity involves the recognition of several PAMPs exhibited by pathogen. Many receptors recognize components of the fungal cell wall leading to the phagocytosis of the fungal cell (Netea and Marodi, 2010; Gow et al., 2012). Recently, it has been described that *C. albicans* grown in the presence of lactic acid can resist to phagocytosis better than when grown in glucose (Ene et al., 2013). This work reported that lactate grown *C. albicans* cells were more efficient at killing and escaping from macrophages (Ene et al., 2013). Given the major impact of carbon source on *C. albicans* cell surface observed in some studies (Ene et al., 2012a,b), the differences reported in terms of phagocytosis may reflect these alterations in cells surface, where PAMPs are present. Therefore, we analyzed the interaction of macrophages and *C. glabrata* cells grown in two different conditions: RPMI and RPMI with acetic acid. We show that acetate-grown *C. glabrata* cells are better phagocytosed than cells grown in glucose (**Figure 3**). Moreover, after 3 h of post-infection, a higher percentage of *C. glabrata’* killing is observed when cells are grown in acetic acid medium than when they are grown in glucose sole medium (**Figure 4**). The presence of acetic acid is not an advantage for fungal cells to deal with macrophages defense, which is in contrast with other reports for *C. albicans* cells grown in the presence of lactic acid. Future studies will compare the effects of lactate and acetate on *C. glabrata* cells and examine the basis for the different effects of these carboxylic acids.

We show that in both suspended cells and biofilm structures, *ADY2a, ADY2b, FPS1*, and *ATO3* genes are more expressed in the presence of acetic acid (**Figure 2**). These results support the notion that these genes may be involved in acetic acid utilization of this carbon-source. We also observed that *ADY2a* and *FPS1* are the most highly upregulated genes under all the conditions tested (**Figure 2**). The expression of these genes seems to vary according to the concentration of fluconazole. In RPMI biofilm cells with or without acetic acid, *FPS1* expression highly increases up to 312.5 μg/ml fluconazole. A high fluconazole concentration however leads to a basal level of expression for all genes, both in the presence and absence of acetic acid, although viable cells are still found in these conditions. These genes seem to be involved in the differential response of cells both to acetic acid and fluconazole.

In *S. cerevisiae* acetic acid can serve as an alternative carbon source to fuel gluconeogenesis via the glyoxylate cycle (Turcotte et al., 2010). As previously mentioned, some studies have recognized the significance of the glyoxylate cycle in the virulence of *C. albicans* (Ramirez and Lorenz, 2007) and *C. glabrata* (Kaur et al., 2007; Fukuda et al., 2013) and have shown that *ADY2* and *FPS1* are expressed inside macrophages (Kaur et al., 2007; Seider et al., 2011).

The phagocytosis assays here reported (**Figure 3**) reveal that macrophages are able to recognize/internalize more efficiently *fps1* and *ady2a* cells than WT cells, when cells are grown in RPMI, indicating that mutant’s cell walls may present some alterations that render them more easily recognized. Beese-Sims et al. (2012) demonstrated that *fps1* was more susceptible to caspofungin, an antifungal agent that targets cell wall glucans. However, in this work we show that *fps1* and WT acetic acid grown cells are more easily recognized/internalized than cells grown in media without the acid. In contrast, the presence of the acid does not affect the interaction of *ady2a* cells with the phagocytes. This suggests that acetic acid does not induce a significant alteration in the cell wall of *ady2a* cells in contrast to what happens in *fps1* and WT cells.

Macrophages are important cells for pathogens’ elimination, as well as immune modulators, presenting antigens and secreting pro-inflammatory cytokines. In this sense we evaluated the macrophages killing of the different *C. glabrata* cells grown in RPMI with or without acetic acid (**Figure 4**). No significant differences are observed in the amount of TNF-α released after incubation, indicating that all cells and conditions are able to similarly activate a pro-inflammatory response. Macrophages are able to kill *fps1* and *ady2a* mutants more efficiently than WT ones (**Figure 4**). However, in cells grown in RPMI and acetic acid, *ady2a* cells are much more refractory to killing when compared to WT cells. Of note, previous studies reported an *ADY2a* upregulation in *C. glabrata* cells upon macrophage phagocytosis (Kaur et al., 2007; Seider et al., 2011). In this work, when *ady2a* cells are grown in RPMI medium, they are more efficiently internalized and phagocytized by macrophages than WT cells. Additionally, when grown in the presence of acetic acid they are more resistant to macrophage killing than WT cells. This suggests a potential role of Ady2a in phagocytosis and reveals that the carbon source clearly affects the cells response to macrophage killing.

## Conclusion

This study shows that acetic acid influences *C. glabrata* behavior in biofilm formation, antifungal drug resistance and phagocytosis. It also suggests that Fps1 and Ady2a might play a role in these processes. Our data support the view that adaptative responses of *Candida* cells to the types of carbon source present in host niches affects the virulence of these fungal cells through multifarious mechanisms (Brown et al., 2014).

## Author Contributions

MC, MH and SP designed the experiments. SM, RA, CC, SS, and SP carried out the experiments. FI and KK provided the mutant strains. SM, AB, KK, PS, MC, MH, and SP analyzed and interpreted the data. SM and SP wrote the manuscript with additional input from RA, CC, SS, AB, KK, PS, MC, and MH.

## Conflict of Interest Statement

The authors declare that the research was conducted in the absence of any commercial or financial relationships that could be construed as a potential conflict of interest.
