# Supplementary material for: Candida glabrata susceptibility to antifungals and phagocytosis is modulated by acetate
Source: Front Microbiol. 2015 Sep 4;6:919. doi: 10.3389/fmicb.2015.00919 (PMC4560035; doi:10.3389/fmicb.2015.00919)
Supplement: Supplementary file 1 [file Data_Sheet_1.DOCX]

***Supplementary Material***

# *Candida glabrata* susceptibility to antifungals and phagocytosis is modulated by acetate

**Sandra Mota^1, 2^, Rosana Alves^1^, Catarina Carneiro^1^, Sónia Silva^3^, Alistair J Brown^4^, Fabian Istel^5^, Karl Kuchler^5^, Paula Sampaio^1^, Margarida Casal^1^, Mariana Henriques^3^ and Sandra Paiva^1*^**

^1^ Centre of Molecular and Environmental Biology, Department of Biology, University of Minho, Braga, Portugal

^2^ Centre of Health and Environmental Research, School of Allied Health Sciences, Polytechnic Institute of Porto, Porto, Portugal

^3^ Centre for Biological Engineering, University of Minho, Braga, Portugal

^4^ Institute of Medical Sciences, School of Medical Sciences, University of Aberdeen, Aberdeen, United Kingdom

^5^ Max F. Perutz Laboratories, Department of Medical Biochemistry, Medical University of Vienna, Vienna, Austria

*** Correspondence:** Dr. Sandra Paiva, Centre of Molecular and Environmental Biology, Department of Biology, University of Minho, Campus de Gualtar, 4710-057 Braga, Portugal.

[spaiva@bio.uminho.pt](mailto:spaiva@bio.uminho.pt)

1. **Supplementary Data**

**Table S1** Primer efficiencies.

| Gene | Best temperature (ºC) | Efficiency |
| --- | --- | --- |
| *ADY2a* | 58.2 | 1.920 |
| *ADY2b* | 58.2 | 2.254 |
| *FPS1* | 57.2 | 1.985 |
| *FPS2* | 56.6 | 2.868 |
| *ATO3* | 58.3 | 2.184 |
| *PGK* | 55 | 1.905 |

**Table S2** CT values used to calculate the relative gene expression in planktonic cells.

| Media condition | Gene | Fluconazole (µg/ml) | CT (mean) | CT (SD) |
| --- | --- | --- | --- | --- |
| RPMI | *ADY2a* | 0 | 18.24 | 0.35 |
|  |  | 50 | 26.58 | 0.17 |
|  | *ADY2b* | 0 | 25.23 | 0.87 |
|  |  | 50 | 29.68 | 0.43 |
|  | *FPS1* | 0 | 21.86 | 0.11 |
|  |  | 50 | 26.69 | 0.39 |
|  | *FPS2* | 0 | 25.53 | 0.25 |
|  |  | 50 | 31.37 | 0.21 |
|  | *ATO3* | 0 | 22.59 | 0.74 |
|  |  | 50 | 29.91 | 2.24 |
|  | *PGK* | 0 | 22.88 | 0.17 |
|  |  | 50 | 30.91 | 0.73 |
| RPMI acetic acid | *ADY2a* | 0 | 18.36 | 0.03 |
|  |  | 50 | 26.41 | 0.64 |
|  | *ADY2b* | 0 | 22.90 | 0.62 |
|  |  | 50 | 28.43 | 0.16 |
|  | *FPS1* | 0 | 21.52 | 0.11 |
|  |  | 50 | 26.35 | 0.38 |
|  | *FPS2* | 0 | 25.49 | 0.58 |
|  |  | 50 | 30.81 | 0.28 |
|  | *ATO3* | 0 | 22.56 | 0.04 |
|  |  | 50 | 27.36 | 0.26 |
|  | *PGK* | 0 | 25.18 | 0.43 |
|  |  | 50 | 28.39 | 0.72 |

**Table S3** CT values used to calculate the relative gene expression in biofilm cells.

| Media condition | Gene | Fluconazole (µg/ml) | CT (mean) | CT (SD) |
| --- | --- | --- | --- | --- |
| RPMI | *ADY2a* | 0 | 16.66 | 0.23 |
|  |  | 312.5 | 25.90 | 1.11 |
|  |  | 1250 | 29.01 | 0.68 |
|  | *ADY2b* | 0 | 25.44 | 0.42 |
|  |  | 312.5 | 29.21 | 0.90 |
|  |  | 1250 | 30.02 | 0.26 |
|  | *FPS1* | 0 | 20.94 | 0.61 |
|  |  | 312.5 | 25.72 | 0.29 |
|  |  | 1250 | 29.85 | 0.04 |
|  | *FPS2* | 0 | 23.28 | 0.43 |
|  |  | 312.5 | 30.30 | 0.37 |
|  |  | 1250 | 32.74 | 0.25 |
|  | *ATO3* | 0 | 20.97 | 0.22 |
|  |  | 312.5 | 28.69 | 1.02 |
|  |  | 1250 | 29.95 | 0.31 |
|  | *PGK* | 0 | 20.77 | 0.61 |
|  |  | 312.5 | 29.98 | 0.70 |
|  |  | 1250 | 27.86 | 0.03 |
| RPMI acetic acid | *ADY2a* | 0 | 17.18 | 0.80 |
|  |  | 312.5 | 25.88 | 0.86 |
|  |  | 1250 | 31.95 | 1.87 |
|  | *ADY2b* | 0 | 22.81 | 0.26 |
|  |  | 312.5 | 26.54 | 0.13 |
|  |  | 1250 | 30.25 | 0.07 |
|  | *FPS1* | 0 | 21.05 | 0.15 |
|  |  | 312.5 | 25.30 | 0.26 |
|  |  | 1250 | 29.00 | 0.02 |
|  | *FPS2* | 0 | 24.94 | 0.61 |
|  |  | 312.5 | 29.76 | 0.07 |
|  |  | 1250 | 32.50 | 0.61 |
|  | *ATO3* | 0 | 22.81 | 0.36 |
|  |  | 312.5 | 28.73 | 0.27 |
|  |  | 1250 | 31.33 | 0.76 |
|  | *PGK* | 0 | 23.54 | 0.58 |
|  |  | 312.5 | 30.16 | 0.97 |
|  |  | 1250 | 30.09 | 0.69 |


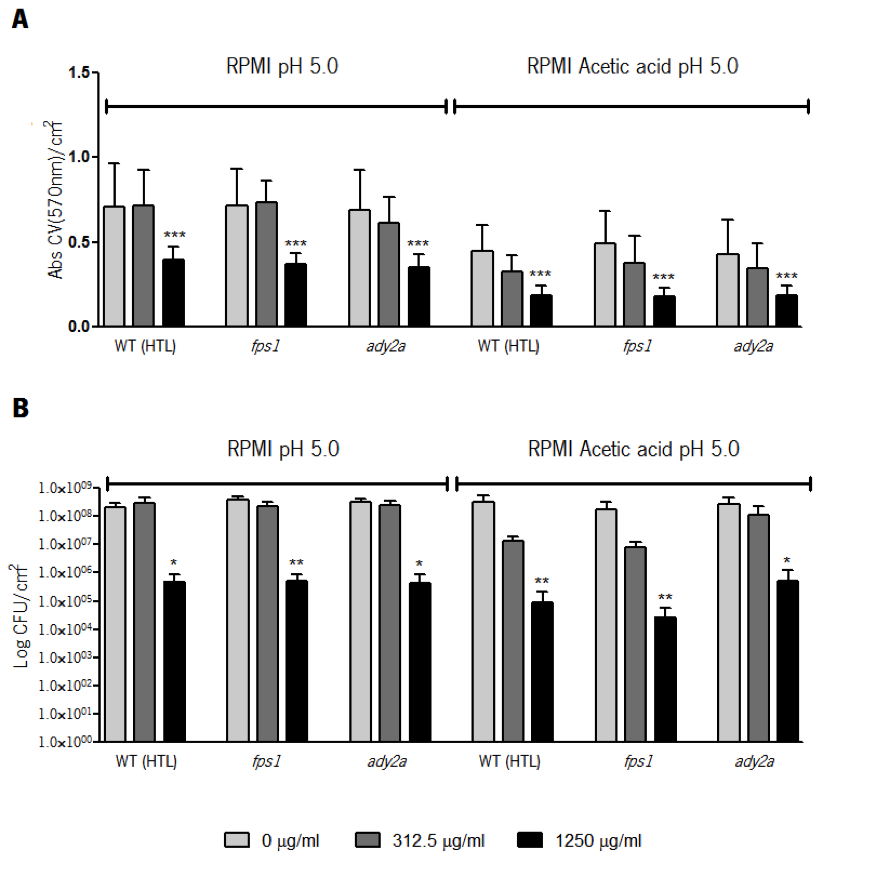


Figure S1. Effect of fluconazole in *C. glabrata* pre-formed biofilms of wild type and *ady2a* and *fps1* mutant cells. Legend displays the different fluconazole concentrations used. Graph A represents the absorbance value of Cristal Violet solution (Abs CV) at 570 nm normalized by unit of area (Abs570 nm /cm^2^) and graph B the logarithm of colony forming units normalized by unit of area (Log_10_ CFU/cm^2^) of biofilm. Two conditions were tested: RPMI pH 5.0 and RPMI 0.5% acetic acid at pH 5.0. Error bars represent standard deviation. *, ** and *** means that results are statistically significant (P<0.05), (P<0.01) and (P < 0.001) respectively.


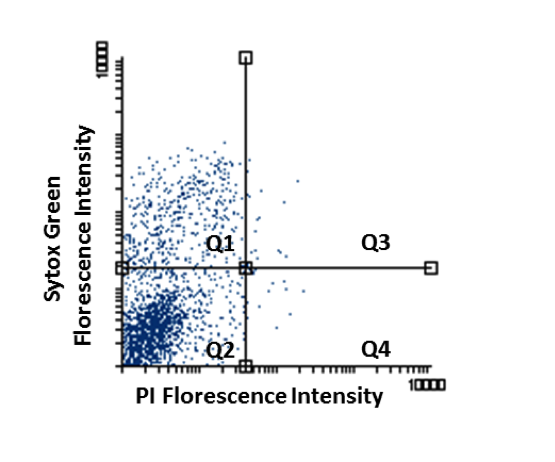


**Figure S2.** Example of cytometry analysis for phagocytosis of *C. glabrata* HTL cells grown in the presence of acetic acid. This figure allows for the identification of macrophages with yeast cells internalized in quadrant Q1 (Sytox Green positive); macrophages without interaction with yeast cells in quadrant Q2 (Sytox Green and PI, both negative); macrophages with ingested and adhered yeast cells in quadrant Q3 ((Sytox Green and PI, both positive), and macrophages with only adhered cells in quadrant Q4 (PI positive).


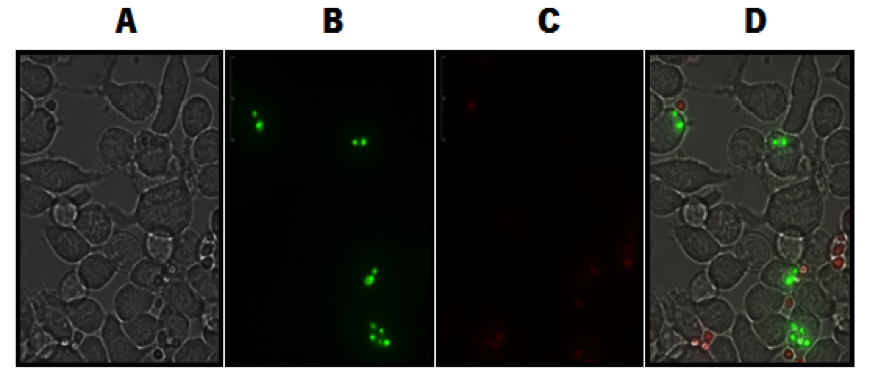


Figure S3. Example of phagocytosis assay observed by fluorescence microscopy. *Candida* cells were labeled with Sytox Green and incubated with macrophages. After 30 min of macrophage infection, cells were washed and stained with propidium iodide. Propidium iodide quenches the SytoxGren and it is excluded from live macrophages, so cells that have undergone phagocytosis remain with green fluorescence, while cells that contact PI are quenched and thus red. This photomicrograph is a portion of a representative field from a phagocytosis assay with *C. glabrata* cells and illustrates the distinct color between extracellular (red) and intracellular (green) cells. A, phase contrast photomicrograph, B green fluorescence photomicrograph, C red epifluorescence photomicrograph and D merge image of B and C fluorescence.
